# Face Database Protection via Beautification with Chaotic Systems

**DOI:** 10.3390/e25040566

**Published:** 2023-03-25

**Authors:** Tao Wang, Yushu Zhang, Ruoyu Zhao

**Affiliations:** College of Computer Science and Technology, Nanjing University of Aeronautics and Astronautics, Nanjing 211106, China

**Keywords:** database protection, adversarial, beautification, chaotic systems

## Abstract

The database of faces containing sensitive information is at risk of being targeted by unauthorized automatic recognition systems, which is a significant concern for privacy. Although there are existing methods that aim to conceal identifiable information by adding adversarial perturbations to faces, they suffer from noticeable distortions that significantly compromise visual perception, and therefore, offer limited protection to privacy. Furthermore, the increasing prevalence of appearance anxiety on social media has led to users preferring to beautify their faces before uploading images. In this paper, we design a novel face database protection scheme via beautification with chaotic systems. Specifically, we construct the adversarial face with better visual perception via beautification for each face in the database. In the training, the face matcher and the beautification discriminator are federated against the generator, prompting it to generate beauty-like perturbations on the face to confuse the face matcher. Namely, the pixel changes produced by face beautification mask the adversarial perturbations. Moreover, we use chaotic systems to disrupt the order of adversarial faces in the database, further mitigating the risk of privacy leakage. Our scheme has been extensively evaluated through experiments, which show that it effectively defends against unauthorized attacks while also yielding good visual results.

## 1. Introduction

The advent of online social networks has revolutionized the way people interact and exchange information in their daily lives. Currently, a growing number of users prefer to share personal images on social networks to express themselves and interact with others. However, the storage of such images in face databases raises privacy concerns. Face images contain information that works as a universal identifier, and automated identification technologies exacerbate the risk of face database leakage, posing a serious threat to users’ privacy. On the one hand, attackers match the leaked images and then rely on the high-performance face recognition system to conduct large-scale monitoring of user behaviors. On the other hand, a large number of soft biometric attributes, e.g., gender, age, and race, contained in face images can be extracted by automatic deep learning-based technologies and then matched with existing databases to further deduce sensitive information about individuals. While restricting the release of face images is a viable solution, it greatly reduces the enjoyment of sharing images by users. Therefore, developing deep learning-based privacy protection techniques is essential to address the problems caused by the technology itself.

To protect the privacy of face databases, one class of methods [1,2,3,4,5,6] aims to generate a reference face to replace the original face, thus completely removing identifiable information. CIAGAN [5] removes the entire face and then generates a face of a specified identity using the one-hot code of another identity. To maintain some utility, CIAGAN only retains some features for face detection. Considering that some scenarios such as forensics still require the original image, Cao et al. [6] introduces a user-specific password to modify the identity features in the spherical space to generate a new face that can be recovered using the password. Even when the password is incorrect, a natural-looking face can still be generated, making it more difficult for an attacker to decrypt. While such methods achieve satisfactory de-identification performance, the significant difference between the visual appearance of the generated face and the original face renders it impossible for the friends to visually recognize the identity of the face in the image, thus defeating the original intention of users to upload their images.

**Figure 1:**
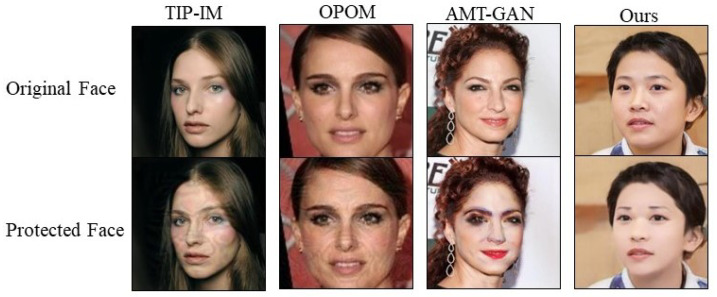
The visual results of our scheme and existing mainstream schemes. The results of TIP-IM and OPOM have obvious perturbations and poor visual effects. The results of AMT-GAN have thick makeup and are visually unnatural. The results of our scheme are natural and have the effect of whitening and thinning the face, which is favored by users.

To avoid the above problem, another class of methods [7,8,9,10,11,12] adds adversarial perturbations to the facial region to evade detection by unknown recognition systems without blocking human perception of identity. TIP-IM [7] allows users to wear an adversarial privacy mask for faces before sharing their images, preventing unauthorized face recognition systems from identifying them while remaining visually identical to the original face for humans. TIP-IM constructs the mask iteratively by optimizing the loss of identity protection and the loss of naturalness, ensuring that the original identities can be concealed without sacrificing the visual quality. Considering that image-specific privacy masks are not only time-consuming but also less secure, OPOM [8] generates person-specific universal masks by optimizing each training sample in the direction away from the feature subspace of the source identity. Various modeling methods are investigated to obtain a better description of the feature subspace, including affine hulls, class centers, and convex hulls. The resulting mask can be added to all images of one user, thus further improving the real-time and practicality of privacy protection. Nevertheless, as shown in Figure 1, the protected faces generated by TIP-IM and OPOM still exhibit noticeable perturbations that can be perceived by human visual systems, detracting from the visual enjoyment of users. To alleviate the unnatural visual effects caused by perturbations, AMT-GAN [12] synthesizes adversarial faces with a target face makeup and identity by using widely used makeup as a key idea for perturbation layout. However, this method may not be accepted by all users, particularly men who may refuse to appear with makeup due to cultural norms of masculinity.

In general, existing methods of face privacy protection, which either replace faces or add perturbations, have limitations in terms of excessive appearance changes or poor visual perception. How to balance the trade-off between visual perception and appearance changes is the main challenge in our scheme.

**Figure 2:**
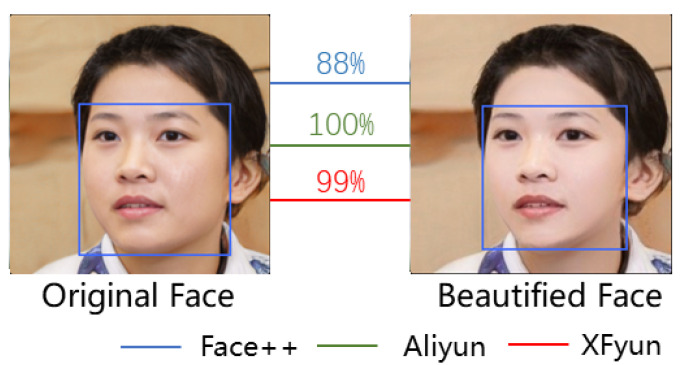
Confidences of identity matching between the beautified face and the original face under different APIs.

Nowadays, short video recording and live-sharing platforms offer the function of automatic face beautification, which is increasingly popular among users who experience appearance anxiety. Users can perform appropriate face beautification before posting selfies on social networks. Unlike makeup that focuses on rendering eye shadow and lip gloss intensely, face beautification [13,14,15] aims at removing skin imperfections and fine-tuning facial features. As a result, appropriate beautification is more acceptable to users as it preserves their natural appearance. It would be of great practical significance if the privacy protection of face databases could be achieved by face beautification. However, as shown in Figure 2, the results generated by existing face beautification techniques focus on preserving the original identity of the face, making the identity of the beautified face more similar to that of the original one. Therefore, face beautification techniques cannot be directly used for database privacy protection.

To this end, this paper considers masking perturbations by face beautification, thus improving the visual naturalness of adversarial faces. Compared to the prior works, our scheme is more visually appealing and has a beautification effect, making it more acceptable to users.

In summary, the main contributions of this paper are as follows:

- We reveal the possibility of protecting face database privacy via beautification, which is more natural than makeup.
- We propose a face database protection scheme via beautification with chaotic systems, which not only alleviates appearance anxiety but also reduces the risk of privacy leakage.
- We set an adversarial threshold for identity loss, preventing facial distortion from excessive deviation of identity features.

## 2. The Proposed Scheme

**Figure 3:**
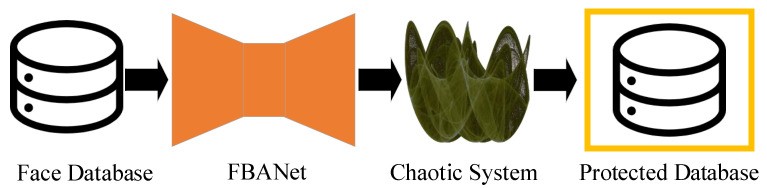
The flow of our scheme for face database protection. Firstly, all the images in the face database are removed from the identifiable information by FBANet. Then, the original order is disrupted by the chaotic system to finally obtain the protected face database.

As shown in Figure 3, the proposed scheme for protecting face databases involves the use of a combination of face beautification through adversarial networks and chaotic systems. The first step in the scheme is to design face beautification adversarial networks (FBANet) that can generate adversarial perturbations. These perturbations can enable face images to evade detection by unknown identifiable machines. To further enhance security, the scheme utilizes a chaotic system to generate random sequences that can disrupt the original order of face images in the database.

### 2.1. Face Beautification Adversarial Networks

FBANet is capable of generating beauty-like perturbations in the full face. In other words, the pixel changes brought by face beautification mask the adversarial perturbations and mitigate the undesirable visual effects caused by the perturbations. In addition, the scheme is able to learn better-generalized features to fight against unknown face recognition systems.

**Figure 4:**
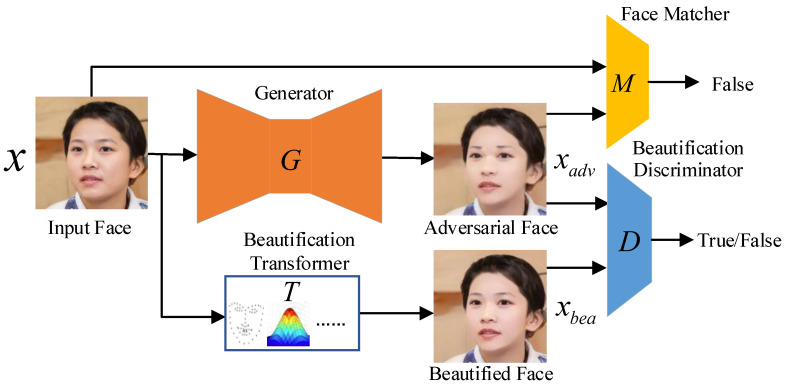
The framework of FBANet. FBANe involves four parts: a generator (G), a beautification transformer (T), a beautification discriminator (D), and a face matcher (M). In the training, T, M, and D are used to assist G to synthesize images that conceal identifiable information while beautifying the face.

As shown in Figure 4, FBANet consists of four parts, which are a generator, a beautification transformer, a beautification discriminator, and a face matcher. Firstly, the input face $x\in R^{H\times W\times 3}$ is input into the beautification transformer to output the beautified face $x_{bea}=T\left(x\right)$, and also input to the generator to generate the adversarial face $x_{adv}=G\left(x\right)$. Then, the face discriminator determines the beautification effect of the beautified face $x_{bea}$ to be true and the adversarial face $x_{adv}$ to be false. The generator performs several rounds of adversarial training with the beautification discriminator, resulting in the adversarial face $x_{adv}$ also having the beautification effect. In addition, the face matcher adds adversarial identity constraints to the adversarial face, so that the generator encourages the identity features of the adversarial face to be different from the original features, thus making the output of the face matcher a mismatch. With the double constraint of the face matcher and the face discriminator on the generator, the generator will eventually generate the adversarial face with the beautification effect.

FBANet is supervised by a weighted sum of four losses: an adversarial beautification loss, an adversarial identity loss, and a reconstruction loss. In the following, we describe the detail of each loss.

#### 2.1.1. Adversarial Beautification Loss

The adversarial loss adopted in generative adversarial networks [16] enhances the sharpness and visual quality of the generated images. As a variant of generative adversarial networks, the purpose of adversarial beautification loss in FBANet is to learn the distribution of beautiful faces, so as to guide the generator to generate face images with strong realism and beautification effects. Meanwhile, the gradient penalty term in WGAN-GP [17] is used to improve the fit of the discriminator to stabilize the adversarial training process. Specifically, the input of the beautification discriminator is a pair of images, and the output is false when the input image pair is $\left(x,x_{adv}\right)$ or true when the input image pair is $\left(x,x_{bea}\right)$. The adversarial losses of the generator and the beautification discriminator are denoted as,

(1) $$L_{adv}^{G}=-E_{x_{adv}}\left[D\left(x,x_{adv}\right)\right]$$

and

(2) $$\begin{array}{c} L_{adv}^{D}=-E_{x}\left[D\left(x,x_{bea}\right)\right]+E_{x_{adv}}\left[D\left(x,x_{adv}\right)\right]+\\ \lambda_{gp}E_{x_{adv}}\left[{\left({∥\nabla_{x_{adv}}D\left(x,x_{adv}\right)∥}_{2}-1\right)}^{2}\right], \end{array}$$

where D(·) outputs the probability that the beautification discriminator predicts whether the input face is a beautified face or not, $E_{x}\left(f\left(x\right)\right)$ denotes the expectation under the distribution f(x) given the conditional variable *x*, and $\lambda_{gp}$ is the coefficient of gradient penalty.

#### 2.1.2. Adversarial Identity Loss

In order to keep the adversarial face from being detected by unauthorized face recognizers, a face matcher is added to supervise the training of the generator so that the output of the adversarial face and the original face is mismatched. The mainstream scheme adopts maximizing the cosine similarity between the original face identity features and the adversarial face identity features to implement the adversarial process with identity loss as,

(3) $$L_{id}=1-E_{x,x_{adv}}\left[M\left(x,x_{adv}\right)\right],$$

where M(·,·) is the similarity of identity features output by the face matcher. Since the above formula fails to control the degree of identity change of the adversarial face, certain regions of the adversarial face will change significantly, such as nose distortion and obvious spots, as the face similarity becomes lower and lower. Considering that existing face recognition systems mostly use a matching threshold to determine whether two faces match or not, it is only required to control the identity similarity below the matching threshold to complete the confrontation. For this reason, we set an adversarial threshold for identity loss. When the identity similarity is lower than the adversarial threshold, the identity loss will no longer decrease. Then, the identity loss is modified as

(4) $$L_{id}=max\left(1-E_{x,x_{ad}}\left[M\left(x,x_{adv}\right)\right],\xi \right),$$

where ξ is the adversarial threshold, which should be smaller than the matching threshold.

In addition, to further improve the transferability of the adversarial faces against attacks from unknown recognition systems, an integrated training strategy that enhances input diversity is used in training. Specifically, the outputs of the joint FaceNet [18] and Cosface [19] recognition models attempt to approximate the decision boundary of the unknown target model. The input diversity is enhanced by adjusting the size of the images and by adding a certain amount of Gaussian noise as a variation function of the inputs. Therefore, the identity loss is further modified as

(5) $$\begin{array}{cc} L_{id}= & \left(1-\alpha \right)\times max\left(1-E_{x,x_{adv}}\left[M_{F}\left(x,T\left(x_{adv}\right)\right)\right],\xi \right)+\\ \; & \alpha \times max\left(1-E_{x,x_{adv}}\left[M_{C}\left(x,T\left(x_{adv}\right)\right)\right],\xi \right) \end{array},$$

where $M_{F}$ and $M_{C}$ represent the outputs of FaceNet and Cosface, T(·) represents the variation function, α controls the extent to which integrated models affect the results.

#### 2.1.3. Reconstruction Loss

The addition of a reconstruction loss serves two main purposes: preserving more details of the original image and enabling better face beautification. Specifically, the mean absolute error of the face and the adversarial face are utilized:

(6) $$L_{rec}={∥x_{bea}-x_{adv}∥}_{1}.$$

#### 2.1.4. Overall Objective

Overall, the objective for the FBANet is formulated as follows:

(7) $$\min_{G}\max_{D}=L_{adv}^{G}+L_{adv}^{D}+\lambda_{1}L_{id}+\lambda_{2}L_{rec},$$

where $\lambda_{i}$ is the hyperparameter for balancing these losses.

### 2.2. The Chaotic System

We apply the chaotic system [20] to disturb the original order of the adversarial images in the database, thus further improving the security of our scheme. The applied chaotic system can be formulated as follows:

(8) $$\left\{\begin{array}{c} {\dot{x}}_{1}=\alpha \left(x_{2}-x_{1}-g\left(x_{1}\right)\right)\\ {\dot{x}}_{2}=x_{1}-x_{2}+x_{3}\\ {\dot{x}}_{3}=-\beta x_{2} \end{array},\right.$$

where $g\left(x_{1}\right)=bx_{1}+0.5\left(a-b\right)\left(\left|x_{1}+1\right|-\left|x_{1}-1\right|\right)$ and α=9.2156, β=15.9946, a=−1.24905, b=−0.75735.

The chaotic system used in our proposed scheme possesses several beneficial properties. Firstly, it exhibits ergodicity, which means that the orbit of a chaotic motion passes through every point within the chaotic interval in a finite amount of time. Secondly, the system displays sensitive dependence on initial conditions, implying that even small deviations from the initial conditions of the system can result in significant differences in the final state. This property makes the long-term evolutionary behavior of chaotic systems unpredictable. Lastly, the system is bounded, indicating that despite the apparent chaos within the system, its trajectory remains confined to a finite region. These properties contribute to improving the security of our scheme.

Concretely, the database disruption is illustrated in Algorithm 1. We utilize the chaotic system to obtain new positions for each image within the database and later disrupt the database according to the new positions.
**Algorithm 1** Database Disruption.**Input:** The face database, *D*; The key, *K*; The number of images in face database, *N***Output:** The disrupted face database, *D*; 1:Using *K* to initialize the chaotic system; 2:O = {}; //recording the new positions of images in the database. 3:**for** i=1 to *N* **do** 4:    Call chaotic system to produce random steam *t*; 5:    t=t mod *N*; 6:    *O* = *O* ∪ {(i,t)}; 7:**end for** 8:Disrupt *D* according to the order of *O*; 9:**return** *D*.

### 2.3. Network Architecture of FBANet

#### 2.3.1. Generator

**Figure 5:**
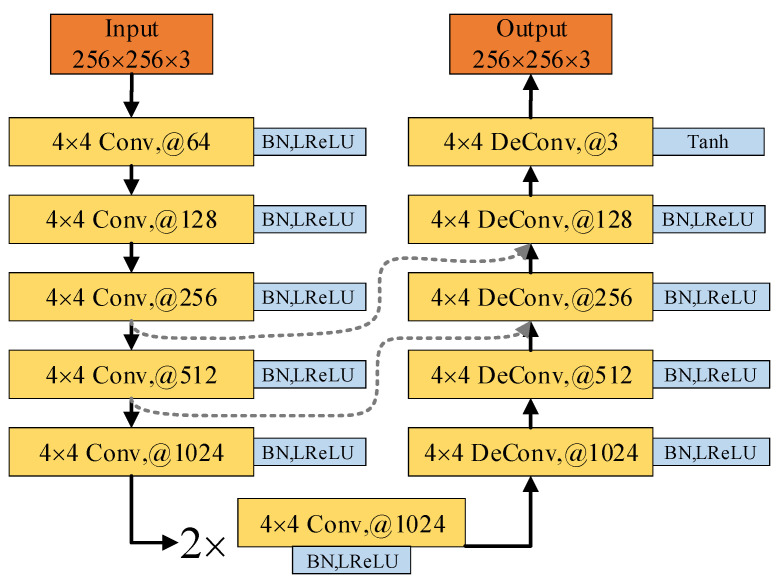
Network structure of the generator. All the convolution layers and deconvolution layers set 4 as kernel size and 2 as stride. BN is batch normalization and LReLU is the LeakyLU layer. The gray dotted arrows are jump connections.

The network structure of the generator is shown in Figure 5, which references the architecture of the autoencoder [21] to efficiently process the original face in the high-level feature layer, making the generated adversarial face more natural. Specifically, the generator receives an input face image of size 256×256×3. The input image undergoes five sequential convolutional layers (Conv) with kernel size 4 and step size 2 for downsampling to obtain high-level features of size 8×8×1024; the high-level features are downsampled by five transposed convolutional layers (DeConv) with a kernel size of 4 and step size of 2 to finally output 256×256×3 size of the adversarial face image. In this case, a batch normalization layer (BN) and a LeakyReLU activation layer (LReLU) are added after each convolutional layer to stabilize and accelerate the training of the model. In particular, only the Tanh activation function is added to the last transposed convolutional layer to map the features to RGB images. In addition, the jump connection layers from U-Net [22] are added between the convolutional and transposed convolutional layers to alleviate the gradient disappearance problem and network degradation during deep network training, while preserving the low-level features such as color and contour output from the intermediate layers of the network.

#### 2.3.2. Beautification Transformer

The beautification transformer converts original faces into beautified faces by eliminating skin imperfections and fine-tuning the proportions of features. Existing mature beautification technologies employ deep learning-based methods to detect faces and locate key points of faces, and then apply computer graphics to achieve face beautification. Our scheme adopts the automatic face beautification API of Face++ as the beautification transformer.

Considering that the high-intensity beauty will obviously change the appearance of the original face and make the original identity unrecognizable by human visual systems, our scheme fixes the relevant parameters of the beautification transformer. Taking the Face++ API used in this scheme as an example, the degree of whitening, the degree of peeling, the degree of slimming, and the degree of large eyes are all set to 50%.

#### 2.3.3. Beautification Discriminator

The beautification discriminator is required to determine that the adversarial face does not have a beautification effect but the beautified face has. Unlike traditional discriminators that use fully connected layers to output classification results as true or false, PatchGAN [23] is designed to output classification results for each perceptual field in the form of full convolution, which focuses more on the local structure and detailed features of the face and will be more conducive to the evaluation of the beauty effect. Therefore, PatchGAN is chosen as the beautification discriminator.

#### 2.3.4. Face Matcher

Consistent with most of the work on face privacy protection, pre-trained face matchers are used as auxiliary models to facilitate the training of the generators. Since the target adversary to be fought against is an unknown recognition model, two advanced face matchers, FaceNet [18] and Cosface [19], both of which have high accuracy on public face image datasets, were selected to learn more generalized features by integrating their prediction results to fight against unknown recognition models.

## 3. Results

### 3.1. Setup

#### 3.1.1. Dataset

Due to the different standards of beautification for different races, our experiment was conducted only for the East Asian race. The Seepretyface dataset was used for training and testing, which contains 50,000 images and covers faces of different genders, ages, and facial attractiveness. The original image resolution was 1024 × 1024, and 10,000 images from the dataset were selected for training and 1000 images for testing.

#### 3.1.2. Implementation Details

The experiments were conducted using the PyTorch deep learning framework and computed by the NVIDIA GeForce RTX 3080 GPU. FBANet was trained with the Adam optimizer, using $\beta_{1}=0.5$ and $\beta_{2}=0.99$. Training images were resized to 256 × 256 resolution, with a batch size of 4 and an initial learning rate of 0.0002. During training, we initially did not include the identity loss and set $\lambda_{2}$ to 100, allowing the generator and discriminator to train alternately. We perform one generator update after five discriminator updates. Once the model’s training was stable, we added the identity loss and set $\lambda_{3}$ to 2, training for a small number of batches.

#### 3.1.3. Baseline

We selected three typical adversarial face generation schemes, including PGD [24], FGSM [25] and GAP [26]. PGD and FGSM find the best perturbation based on iterative optimization, while GAP learns the generalized perturbation to each image using a GAN model, similar to our scheme. In addition, their original classification loss function is replaced with the identity feature loss, and FaceNet is chosen as the white-box face recognition classifier they need to defend.

### 3.2. Selection of Adversarial Thresholds

**Figure 6:**
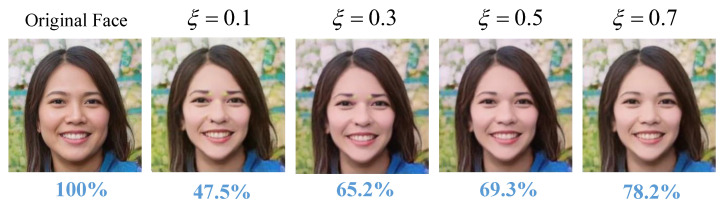
Adversarial faces with different adversarial thresholds and the corresponding confidence levels blue marked.

The adversarial threshold is used to prevent distortion of the face region due to excessive deviation of identity features. Setting the threshold too high preserves most of the identity information, resulting in poor privacy protection, while setting it too low results in severe loss of identity information and poor visual quality. Since the adversarial threshold needs to be smaller than the matching threshold (0.75), the value of 0.2 is taken from 0.1 to 0.7 interval as the adversarial threshold to carry out the corresponding experiment. The results are shown in Figure 6, where the blue numbers indicate the similarity between the adversarial face and the original face. At the adversarial threshold of 0.1, the nose of the face is distorted on both sides, while other color spots appear around the eyes. When the value is taken as 0.5, the adversarial face can both maintain high visual quality and effectively reduce the identity similarity. Although the result is visually better at the adversarial threshold of 0.7, privacy cannot be protected because the confidence level is greater than 0.75. Therefore, 0.5 is chosen as the adversarial threshold in our scheme.

### 3.3. Visual Evaluation

**Figure 7:**
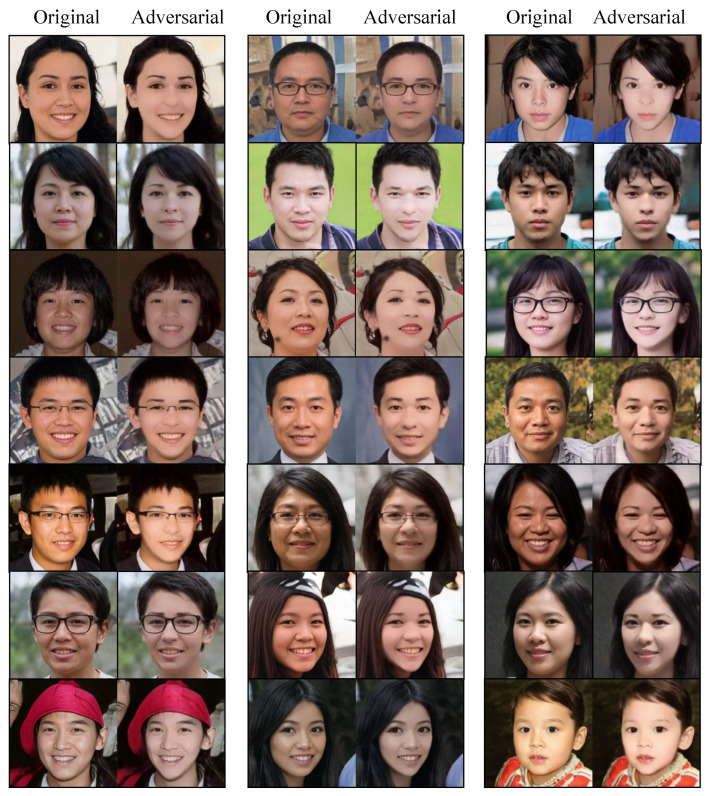
The adversarial face images generated by our scheme.

Figure 7 shows the results generated by FBANet on the Seepretyface yellow dataset, where the first, third, and fifth columns are the original images, and the second, fourth, and sixth columns are the corresponding adversarial images, respectively. It can be observed that FBANet is capable of face beautification, including peeling, whitening, and face-slimming effects. Compared to the original face, the face generated by FBANet has more delicate features and fairer skin.

**Figure 8:**
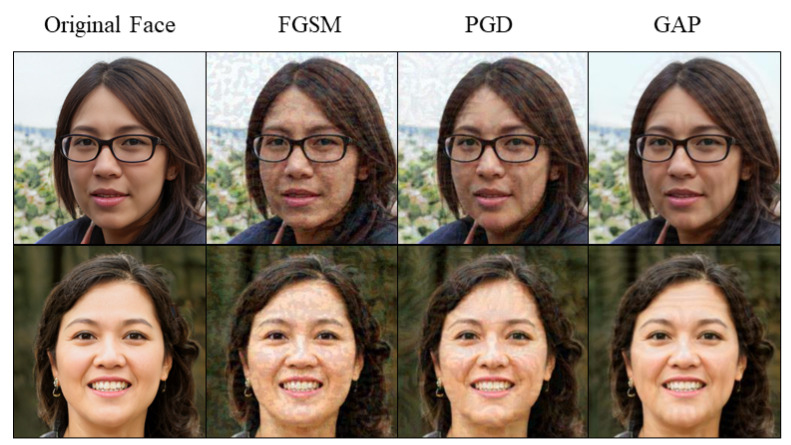
Adversarial images generated by the benchmark schemes.

Figure 8 shows the results generated by the benchmark scheme. It is clear that there are different shapes of perturbation information on the adversarial face, whereas there is also perturbation information in the non-face region, which affects the visual experience of users and is not favorable for image sharing in social networks.

### 3.4. Evaluation of Privacy Protection

To evaluate the effectiveness of the proposed scheme for privacy protection, we test using five face recognition models, namely FaceNet [18], CosFace [19], ArcFace [27], SENet [28], and MobileFace [29]. Successful privacy protection is defined when the identity feature similarity of the original face is less than the identity matching threshold, i.e., $I(x,\hat{x})<\delta$ where *x* is the original face, $\hat{x}$ is the adversarial face, and δ is the matching threshold, which is set to 0.75 in our experiment. The successful protection rate (SPR) is used as the criterion for privacy protection evaluation.

(9) $$SPR=\frac{1}{m}\sum_{i=1}^{m}sp\left(x_{i}\right)$$

where *m* is the number of test samples and

(10) $$sp\left(x\right)=\left\{\begin{array}{c} 0,I(x,\hat{x})>\delta \\ 1,I(x,\hat{x})\leq \delta \end{array},\right.$$

Table 1 presents the SPRs of the benchmark scheme and our scheme under different face recognition models. The traditional adversarial perturbation methods, i.e., PGD and FGSM, achieved SPRs of 100% for FaceNet, indicating that such iterative optimization methods are most effective for white-box models. Meanwhile, they achieve SPRs of nearly half for SENet and Mobileface. However, their SPRs for CosFace and ArcFace are almost zero, indicating that they cannot resist the attacks of certain unknown models. GAP uses generative adversarial networks to generate adversarial perturbations, and its protective effect is significantly improved compared with PGD and FGSM. It has a better generalization effect on SENet and Mobileface, and it also has a good generalization effect on CosFace and ArcFace. The protection effect is significantly better than that of PGD and FGSM. In contrast, although our scheme cannot achieve the optimal SPRs on all models, it can achieve satisfactory protective effects on all these models and has a stronger generalization effect. In general, the proposed scheme can better resist attacks on face recognition models than the benchmark schemes.

With the development of adversarial samples, some face-matching commercial APIs periodically update their face-matching algorithms so as to resist the attacks of adversarial attacks. It will be more relevant if the designed privacy protection scheme can resist face-matching commercial APIs. Therefore, adversarial experiments were conducted on domestic face-matching commercial APIs.

Table 2 shows the average confidence of the matching results between the faces generated by different schemes and the original face on three domestic commercially available APIs, where a smaller average confidence indicates a lower similarity of the faces, namely, a better privacy protection effect. It is important to note that the matching thresholds for each API are not consistent. As can be seen from Table 2, GAP obtains the highest confidence level and is the least effective in resisting face-matching APIs. That is because it generates partial perturbation in non-face regions as well, but the advanced face-matching APIs only keep face regions for recognition. PGD and FGSM also reduce the face matching confidence, but the effect is not significant. The identity matching confidence of the adversarial face generated by our scheme against the original face reaches the lowest level among all schemes, so the proposed scheme has better privacy protection than the benchmark schemes.

### 3.5. Efficiency Evaluation

Table 3 shows the average time required to generate an adversarial face and the parameters of the model used for the proposed scheme and the benchmark scheme. Both PGD and FGSM are based on an iterative optimization method to generate the adversarial perturbation, and thus take more time compared to GAP, but they do not require model parameters. The proposed scheme needs to consider both face beautification and privacy and thus is more complex in terms of model structure than GAP. Therefore, our scheme has more parameters than GAP. Since the depth model can process the data in parallel with the help of GPU, the difference in time efficiency between the scheme of this paper and GAP is not significant.

### 3.6. Ablation Study

**Figure 9:**
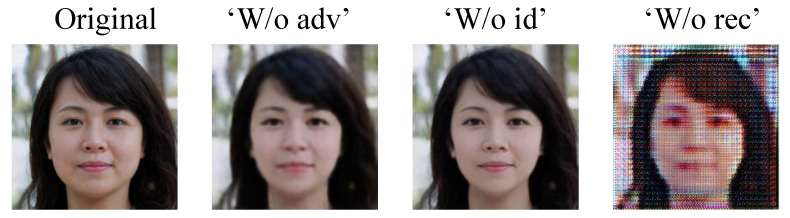
Ablation results.

In this part, we conduct ablation experiments to analyze the individual contributions of the three loss components: adversarial beautification loss, adversarial identity loss, and reconstruction loss. Figure 9 shows the results when excluding one of these losses. Upon removing adversarial beautification loss (‘w/o adv’), the generated image was observed to be slightly blurry, which highlights the significant role played by the adversarial beautification loss in enhancing the visual quality. Similarly, when adversarial identity loss (‘w/o id’) was excluded, the generator solely outputs the beautified face, which maintains the original identity. Lastly, removing reconstruction loss (‘w/o rec’) helps protect the identity, but it resulted in a significant loss of details, thereby reducing the visual fidelity of the protected image.

### 3.7. Discussion

Unlike the benchmark schemes that add perturbations directly to the face image, which is susceptible to detection by adversarial defense methods, the perturbations generated by FBANet operate on the feature level of the identity rather than the pixel level, thus generating more natural and difficult-to-detect adversarial faces. However, with the continuous development of face recognition technology, the perturbations generated by FBANet at the feature level may be removed by face recognition technology, leaving private identity information unprotected.

One limitation of our scheme is that it strictly fixes the beautification parameters, which may not meet the personalized demands of individual users. As such, it may be challenging to apply our scheme in real-world scenarios.

In future work, we will focus on two main areas of improvement. Firstly, we consider improvements in face beautification. Because users have unique standards of beauty, there is a requirement to further provide users with modifiable beautification parameters in order to achieve controllable face beautification. Secondly, we consider improvements in privacy protection. As face recognition technology evolves, the constructed adversarial perturbations may be ignored by adversaries, resulting in identity information remaining compromised. Therefore, constructing transferable, interpretable, and robust adversarial faces is one of the main points we need to focus on.

## 4. Conclusions

The sensitive information contained in face databases is vulnerable to leakages, which raises privacy concerns. The results generated by existing privacy protection schemes are not natural. In this paper, we design a face database protection scheme via beautification with a chaotic system. The scheme uses the face matcher and the beautification discriminator to collaboratively supervise the training process of the generator, prompting it to generate a beauty-like perturbation on the original face. Subsequently, the chaotic system is applied to disrupt the order of faces in the database to obtain the protected database. Adequate experiments conducted on the Seepretyface yellow dataset show that our scheme is not only able to generate more natural faces but also effectively defends attacks by multiple face recognition models and commercial face matching APIs.

## Figures and Tables

**Table 1:** Successful protection rates of different adversarial schemes against face recognition models.

	PGD	FGSM	GAP	Ours
FaceNet	1	1	1	0.846
Cosface	0	0	0.43	0.727
Arcface	0.019	0.09	0.31	0.672
SENet	0.62	0.659	0.85	0.845
Mobileface	0.538	0.59	0.83	0.765

**Table 2:** Confidence levels of different adversarial schemes on commercial APIs for face matching.

	PGD	FGSM	GAP	Ours
Face++	0.861	0.845	0.921	0.761
Aliyun	0.892	0.853	0.999	0.812
XFyun	0.981	0.967	0.999	0.915

**Table 3:** The average time and the model parameter of different adversarial faces schemes.

	PGD	FGSM	GAP	Ours
Average Time	1.593 s	1.015 s	0.1123 s	0.1143 s
Parameters	–	–	7.8 M	43 M

## Data Availability

The dataset can be loaded from http://seeprettyface.com/mydataset.html (accesed on 23 March 2023).
